# Conserved histidine residues and the control of tick-borne encephalitis virus maturation

**DOI:** 10.1099/jgv.0.002286

**Published:** 2026-06-18

**Authors:** Iris Medits-Weiss, Lena Roßbacher, David N. Springer, Franz X. Heinz, Karin Stiasny

**Affiliations:** 1 Center for Virology, Medical University of Vienna, Vienna, Austria

**Keywords:** major envelope protein E, orthoflavivirus maturation, pH sensor, tick-borne encephalitis virus

## Abstract

The maturation and infectivity of orthoflaviviruses is driven by interactions and structural changes of the two envelope proteins, prM and E, which are controlled by protonation and deprotonation events during the viral life cycle when the virus encounters acidic and neutral pH environments inside and outside the cell. Histidine residues act as sensors for these pH-induced conformational transitions, which differ between mosquito-borne and tick-borne orthoflaviviruses in some aspects. We identified six histidine residues that are conserved among the envelope proteins of mammalian tick-borne orthoflaviviruses, but not among those of mosquito-borne orthoflaviviruses. These residues might account for some of the structural differences observed between these two orthoflavivirus groups. We therefore conducted a mutational analysis by replacing each of the conserved histidine residues with alanine and determined the effect of these mutations on the maturation and infectivity of tick-borne encephalitis virus (TBEV). One of the histidine residues (H208, located in an insert unique to mammalian tick-borne orthoflaviviruses at the prM-binding site in E) was shown to be an important determinant of efficient prM cleavage. In line with recent findings regarding the infectivity of immature tick-borne orthoflaviviruses, this mutant was only slightly less infectious than the mature wild-type virus. The presence of histidine residue 208 appears to be linked to the particular requirements of the complex ecological life cycle of TBEV and likely reflects an evolutionary adaptation to the specific interactions of the virus with mammalian hosts and/or tick vectors.

## Introduction

**Fig. 1.**
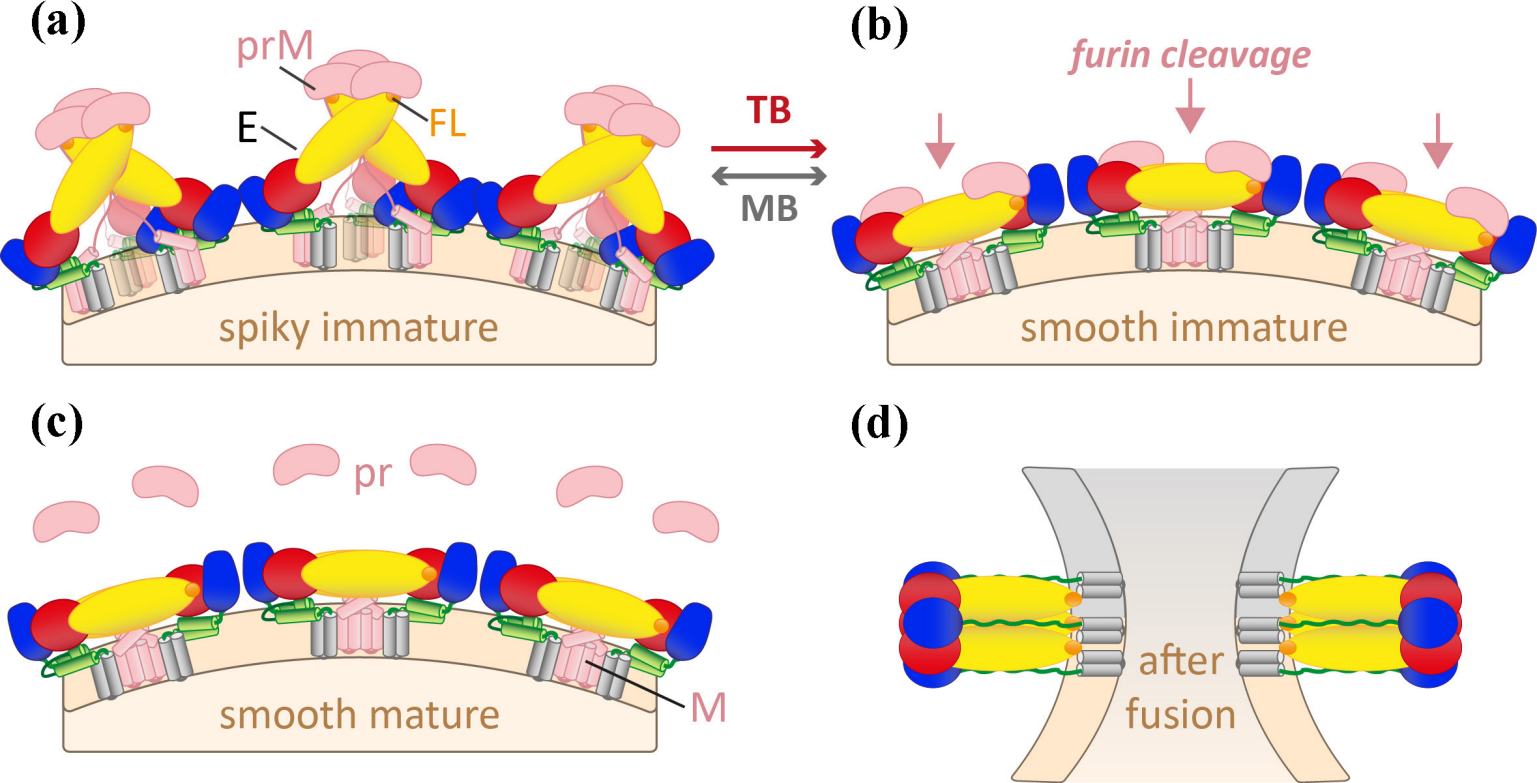
Schematic representation of maturation and entry steps of the cellular life cycle of orthoflaviviruses. (**a**) Trimeric prM–E spikes on the surface of immature virions in the ER (neutral pH). (**b**) prM–E heterodimers on the surface of immature virions in acidic compartments of the secretory pathway, where prM cleavage by furin occurs. The pr fragments stay associated with E at low pH. The acidic pH-induced conformational change allowing furin cleavage site exposure is irreversible in tick-borne (TB) orthoflaviviruses [red arrow between panels (a) and (b)], but reversible in mosquito-borne (MB) orthoflaviviruses [grey arrow between panels (a) and (b)]. (**c**) E homodimers with the membrane-associated M-fragment of prM underneath E on the surface of mature extracellular virions (neutral pH) and dissociated pr. (**d**) Post-fusion E trimer formation and fused membranes in the endosome, triggered by acidic pH after receptor-mediated endocytosis. Colour code E: domain I, red; domain II, yellow; domain III, blue; fusion loop (FL), orange; stem, green; and membrane anchor, grey. prM/M is shown in pink, the viral membrane in beige and the endosomal membrane in grey.

Orthoflaviviruses include several important mosquito-borne and tick-borne human pathogens, e.g. dengue, Zika and West Nile viruses and tick-borne encephalitis virus (TBEV), respectively [1]. These small, enveloped viruses, which form the genus *Orthoflavivirus* in the family *Flaviviridae*, undergo specific, pH-dependent structural changes during their life cycle that control virus assembly, maturation and entry [2]. They assemble by budding as immature particles into the lumen of the endoplasmic reticulum (ER), in which the surface proteins prM (pre-membrane) and E (envelope) form 60 trimeric spikes [3] (Fig. 1a). During maturation and release, virions encounter acidic pH in Golgi subcompartments [4], which induces conformational changes in the envelope protein complex, enabling furin cleavage of prM into pr and M (membrane) and the formation of a smooth herringbone-like icosahedral lattice of the surface proteins [5]. A recent study with tick-borne orthoflaviviruses suggests that furin cleavage may be involved in completing this low-pH-induced switch from the spiky to the smooth conformation [6].

At the acidic pH of compartments of the secretory pathway, the cleaved external pr-part of prM stays associated with the E dimers (Fig. 1b) but is shed upon release into the neutral extracellular milieu, giving rise to fully mature infectious viruses [5] (Fig. 1c). In these mature virus particles, in which E dimers in association with the M protein are arranged in a herringbone-like lattice, the E protein has a metastable conformation and mediates both receptor-binding and membrane fusion [7]. After uptake of mature virions by a target cell through receptor-mediated endocytosis, the acidic pH in the endosome triggers extensive structural changes in E, including the dissociation of the E dimers, exposure of the fusion loop and the formation of a hairpin-like structure of E trimers, which together drive fusion of the viral and endosomal membranes, leading to the release of the genome into the cytoplasm [8] (Fig. 1d).

There is evidence that histidine residues in prM and E conserved across all orthoflaviviruses act as pH-sensors and by their protonation and deprotonation control the structural rearrangements of these proteins during maturation and entry [9,11]. A key difference between tick-borne and mosquito-borne orthoflaviviruses lies in the acidic pH-induced conformational change of prM–E heterodimers that enables prM cleavage during maturation. This change is irreversible for tick-borne orthoflaviviruses (as shown for TBEV and Langat virus [12,13]), but reversible for mosquito-borne orthoflaviviruses, as shown for dengue, West Nile and Usutu viruses [5,13] (Fig. 1). This difference has important consequences for the infectivity of immature viruses. TBEV grown in a furin-deficient cell line (and therefore consisting of completely immature particles) proved to be significantly more infectious in cells and a mouse model than immature mosquito-borne orthoflaviviruses such as West Nile, Usutu and Zika viruses [13]. Apparently, the irreversible conformational change of the TBEV prM–E complex at acidic pH in the secretory pathway leaves the furin cleavage site (FCS) exposed, allowing prM processing also at the cell surface and leading to substantial infectivity of immature virions [13]. In contrast, the reversion of the smooth to the spiky form of immature virions at neutral pH, characteristic of mosquito-borne orthoflaviviruses (Fig. 1), occludes the FCS and renders their immature forms significantly less infectious [13].

As these differences may be an expression of variations in the pH-sensing processes of maturation between these viruses, we investigated histidine residues that are conserved in the envelope proteins of tick-borne orthoflaviviruses, but not in those of mosquito-borne orthoflaviviruses. We found six histidine residues in mammalian tick-borne orthoflaviviruses that met these criteria (one in prM and five in E) and studied their role in virus maturation and infectivity of TBEV. The possible functions of these residues were assessed by carrying out mutational analyses of recombinant subviral particles (SVPs) and infectious virions.

These analyses revealed a mammalian tick-borne orthoflavivirus-specific histidine residue (E-H208), located at the pr-binding site in E in the smooth immature form, as an important determinant for efficient prM cleavage and, consequently, maturation of TBEV. Consistent with recent findings on the infectivity of immature tick-borne orthoflaviviruses, this mutant was only slightly less infectious than the mature WT virus. The other tick-borne orthoflavivirus conserved residues appear to have no direct role in the maturation process.

## Methods

### Phylogenetic analysis and sequence alignment

**Fig. 2.**
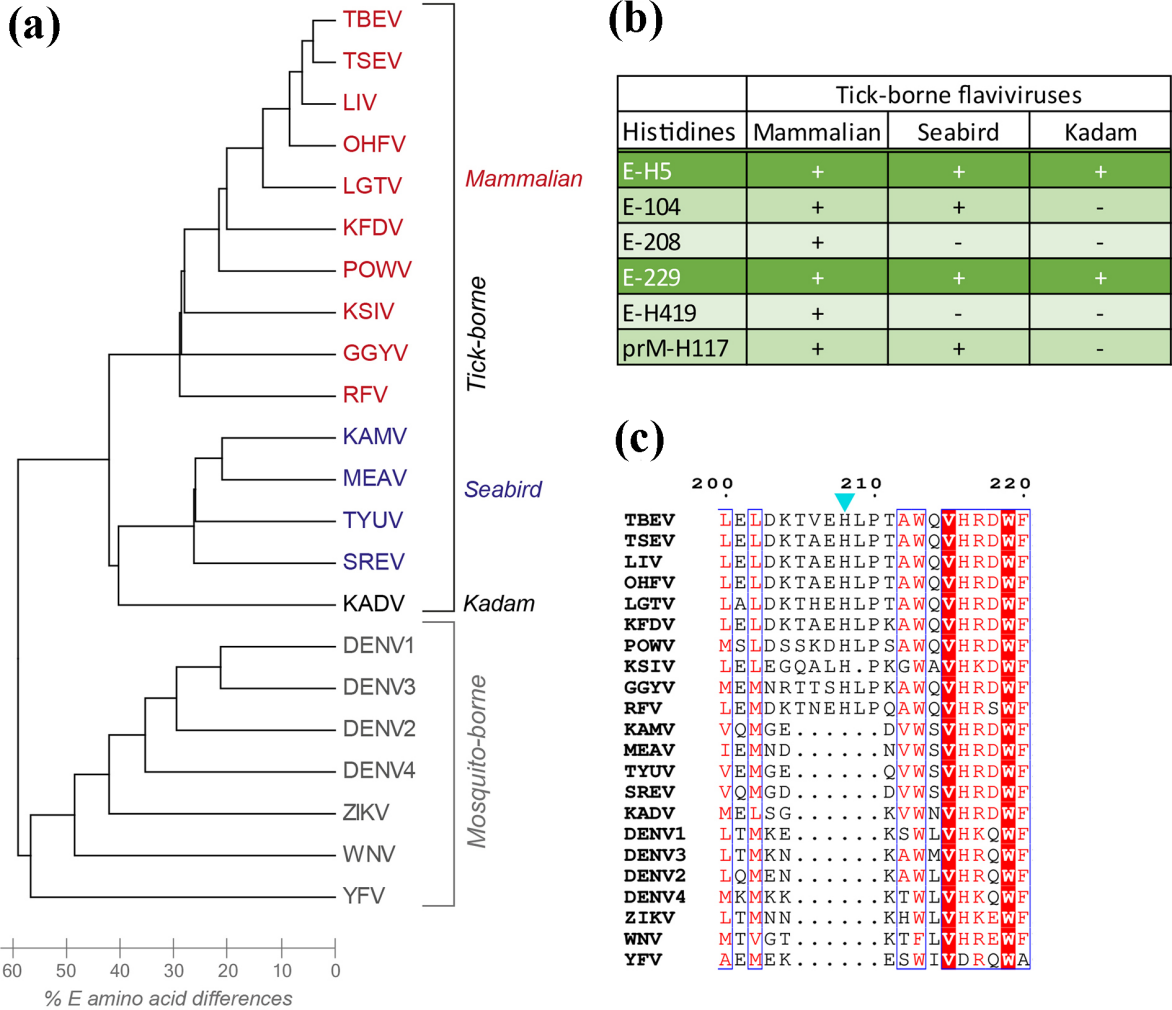
Relationships among orthoflaviviruses and tick-borne conserved histidine residues in prM and E. (**a**) Dendrogram of orthoflaviviruses based on percent amino acid differences in E proteins. (**b**) Conservation of tick-borne-specific histidine residues across the three groups of tick-borne orthoflaviviruses. + indicates the presence and - the absence of a histidine. (**c**) Alignment of TBEV E residues 200 to 220 with E of other tick-borne and mosquito-borne orthoflaviviruses using MAFFT (https://mafft.cbrc.jp) and ESPript (https://endscript.ibcp.fr [15]). E-H208 is indicated by a cyan triangle. TBEV amino acid numbering above the alignment. TSEV, Turkish sheep encephalitis virus; LIV, louping Ill virus; OHFV, Omsk haemorrhagic fever virus; LGTV, Langat virus; KFDV, Kyasanur forest disease virus; POWV, Powassan virus; KSIV, Karshi virus; GGYV, Gadgets Gully virus; RFV, Royal farm virus; KAMV, Kama virus; MEAV, Meaban virus; SREV, Saumarez Reef virus; TYUV, Tyuleniy virus; KADV, Kadam virus; DENV1, dengue virus serotype 1; DENV3, dengue virus serotype 3; DENV2, dengue virus serotype 2; DENV4, dengue virus serotype 4; ZIKV, Zika virus; WNV, West Nile virus; YFV, yellow fever virus.

Amino acid sequences of orthoflavivirus E proteins were retrieved from GenBank using the following accession numbers: TBEV, tick-borne encephalitis virus (U27495); TSEV, Turkish sheep encephalitis virus (DQ235151); LIV, louping Ill virus (NC_001809); OHFV, Omsk haemorrhagic fever virus (AY193805); LGTV, Langat virus (AF253419); KFDV, Kyasanur forest disease virus (AY323490); POWV, Powassan virus (L06436); KSIV, Karshi virus (DQ235147); GGYV, Gadgets Gully virus (DQ235145); RFV, Royal farm virus (DQ235149); KAMV, Kama virus (KF815940); MEAV, Meaban virus (DQ235144); SREV, Saumarez Reef virus (DQ235150); TYUV, Tyuleniy virus (DQ235148); KADV, Kadam virus (DQ235146); DENV1, dengue virus serotype 1 (GQ398255); DENV3, dengue virus serotype 3 (EU081190); DENV2, dengue virus serotype 2 (NC_001474); DENV4, dengue virus serotype 4 (GQ398256); ZIKV, Zika virus (KJ776791); WNV, West Nile virus (DQ211652); and YFV, yellow fever virus (AY640589). Multiple sequence alignments were generated using MAFFT (v7) with default parameters. The phylogenetic relationships shown in Fig. 2(a) are based on these aligned E protein sequences. Evolutionary analyses were conducted in mega X using the Unweighted Pair Group Method with Arithmetic Mean (UPGMA) [14]. The multiple sequence alignment of TBEV E residues 200 to 220 with E of other tick-borne and mosquito-borne orthoflaviviruses was visualized with ESPript (https://endscript.ibcp.fr [15]).

Histidine residues conserved across tick-borne orthoflaviviruses were identified with amino acid alignments of prM and E proteins, comparison of prM and E sequences using the NCBI virus variation (accessed in September 2022; http://www.ncbi.nlm.nih.gov/genomes/VirusVariation/) and previous publications [16,17].

### Mutagenesis of recombinant SVPs

Point mutations were introduced into the pSV-PE WT plasmid encoding the structural proteins prM and E of the European subtype TBEV virus strain Neudoerfl (GeneBank accession number U27495) under the control of the SV40 promoter [18] by site-directed mutagenesis (GeneArt site-directed mutagenesis system from Invitrogen) following the manufacturer’s instructions (Life Technologies). The single mutations and the primers (VBC Biotech and Eurofins Genomics) used for site-directed mutagenesis are listed in Table 1.

**Table 1.** Primers used for site-directed mutagenesis of the pSV-PE WT vector containing prM and E of the TBEV strain Neudoerfl

Mutation	Primer	Sequence (5′→3′)
E-H5A	Fwd	GCTTCGCGTTGCACAGCCTTGGAAAACAGGGAC
	Rev	GTCCCTGTTTTCCAAGGCTGTGCAACGCGAAGC
E-H104A	Fwd	CGAGGCTGGGGCAACGCCTGTGGACTGTTTGGA
	Rev	TCCAAACAGTCCACAGGCGTTGCCCCAGCCTCG
E-H208A	Fwd	GACAAGACAGTGGAAGCCCTTCCAACGGCTTGG
	Rev	CCAAGCCGTTGGAAGGGCTTCCACTGTCTTGTC
E-H229A	Fwd	ATCTGGCTCTGCCATGGAAAGCCGAGGGAGCGCAAAACTGGAA
	Rev	TTCCAGTTTTGCGCTCCCTCGGCTTTCCATGGCAGAGCCAGAT
E-H419A	Fwd	ACAGTGATAGGAGAGGCCGCCTGGGACTTCGGT
	Rev	ACCGAAGTCCCAGGCGGCCTCTCCTATCACTGT
prM-H117	Fwd	GACTCGCTGCGAACAGCCCTTACTAGAGTTGAG
	Rev	CTCAACTCTAGTAAGGGCTGTTCGCAGCGAGTC

### Mutagenesis of cDNA clones

The infectious cDNA clone is based on the European subtype TBEV strain Neudoerfl (GeneBank accession number U27495). The pTNd/c plasmid contains the entire genomic cDNA (sequence 1–11,141 nt) in the pBR322 vector under the control of the T7 transcription promoter [19].

Single mutations were introduced into the pTNd/c plasmid by restriction-free (rf) cloning (overlap extension PCR cloning or ligation independent cloning) as described in [20]. The first PCR with the pSV plasmid (for SVP production) was the template, and a pair of hybrid primers yielded a DNA fragment containing complementary sequences to the region carrying the mutation. The resulting PCR product was purified by preparative agarose gel electrophoresis and used as primers in the second PCR with a methylated pTNd/c plasmid as template. The parental plasmids were degraded with the methylation-sensitive restriction enzyme *DpnI* (New England Biolabs) for 2 h at 37 °C and 20 min at 80 °C [20], and the final product was transformed into 10-beta competent *Escherichia coli* cells (New England Biolabs) according to the manufacturer’s protocol.

After verification of the presence of the mutation by sequence analysis according to Sanger [21], the plasmid DNA was amplified by transformation into electrocompetent HB101 *E. coli* cells (Gibco BRL) as recommended in the manufacturer’s protocol. Primer design was done with the online tool for the design of rf cloning projects (http://www.rf-cloning.org/ [20]). A list of primers for the appropriate exchange is listed in Table 2.

**Table 2.** Primers used in the first PCR to amplify the ‘megaprimers’ for the second PCR

Mutation	Primer	Sequence (5′→3′)
E-H5AE-H104A	Fwd	GCTCCTGTGTTTGGCACCGGTTTACGCTTCGCGTTGCACA
	Rev	GCCGCCTTGACACAGGCCACAATGCTACCCTTTCCAAACAGT
E-H208AE-H229A	Fwd	CGTTGACTTGGCCCAGACCGTCATCCTTGAGCTTGACAAGACA
	Rev	TGTCACACATTGTGTACGTAAGACCTTTCATCTTCAGTTTTTCCAGTCCC
E-H419A	Fwd	GAGTCATCAATGGTTCCAAAAAGGGAGCAGCATCGGAAGGGTTTTCC
	Rev	GTAGAAACCCCACTCCCCCGAAGATGCTGTTGAAAGCGCC
prM-H117A	Fwd	GTGTTTGGCACCGGTTTACGCTTCGCGTTGCACACACTTG
	Rev	ACCCTTGGAGTGGGGGCGGATGTTGGTTGCGCTGTGGACA

### DNA sequence analysis

Sequence analysis was performed using the ABI Prism Big Dye Terminator Cycle Sequencing Kit (Applied Biosystems) in combination with a capillary sequencer GA 3100 (Applied Biosystems) according to the manufacturer’s instructions. The sequences were evaluated with the software Geneious Pro (version 5.0.4).

### Production of recombinant SVPs

African green monkey kidney fibroblast-like cells (COS-1 cells; American Type Culture Collection ATCC no. CRL-1650) were grown in Dulbecco’s modified Eagle’s medium (DMEM) supplemented with 10% FBS and 1% penicillin–streptomycin–glutamine (all from Gibco) at 37 °C.

SVPs were produced as described previously [18,22, 23]. Five microgrammes of WT or mutant DNA was electroporated into COS-1 cells using the GenePulser from Bio-Rad with the following settings: 1.5 kV and 25 µF. Twenty to twenty-two hours after electroporation, the medium was replaced by DMEM (25 mM HEPES, Gibco) without FBS. The supernatant was harvested and cleared by centrifugation (10,000 r.p.m.; 30 min; 4 °C: Beckmann JA 14) 48 h post-electroporation. Immature SVPs were produced as described previously [22]. Twenty to twenty-two hours post-electroporation, the medium was replaced with DMEM (25 mM HEPES, Gibco) without FBS, supplemented with 50 mM NH₄Cl. E protein in the cell culture supernatant was quantified by ELISA, as described below.

### Ultracentrifugation of SVPs

Harvested and clarified cell culture supernatants were applied to a 10% sucrose cushion in TAN (50mM triethanolamine, 100 mM NaCl, pH 8.0) buffer. Ultracentrifugation was carried out for 2 h at 50,000 r.p.m. at 4 °C (Beckmann Ti 90) as described previously [24,25]. The resulting pellets containing the particles were resuspended in TAN buffer pH 8.0 supplemented with 0.1% BSA. E protein in the supernatant and pellet fractions was quantified by ELISA, as described below.

### In vitro transcription of RNA

*In vitro* transcription of WT and mutant cDNA was carried out as described previously [25,26]. Six microgrammes of cDNA was digested with the restriction enzyme NheI (Thermo Fisher Scientific), leaving 5′ overhangs (5′-CTAG). These ends were filled with the Klenow fragment (Thermo Fisher Scientific) to generate blunt ends. After phenol–chloroform purification, cDNA was transcribed using the T7 Megascript transcription kit (Ambion), followed by RNA purification with the RNeasy Cleanup system (Qiagen) according to the manufacturer’s instructions. The concentration was determined with the NanoDrop 2000 spectrophotometer (Thermo Fisher Scientific), and the quality of transcription was controlled by RNA gel electrophoresis [25].

### Production of virus particles from an infectious clone

Baby hamster kidney (BHK-21) cells (ATCC no. CCL-10) were grown in Eagle’s minimum essential medium (MEM, Sigma-Aldrich) supplemented with 5% FBS, 1% l-glutamine and 0.5% neomycin at 37 °C. A total of 7.8 µg *in vitro* transcribed RNA was electroporated into BHK-21 cells (1.2×10^6^ cells ml^−1^) using the GenePulser from Bio-Rad with the following settings: 1.8 kV, 25 µF and 200 Ω. After electroporation, cells were seeded into a 75 cm² tissue culture flask, and 6 h post-electroporation, the medium was replaced by MEM 1% FBS, 1% l-glutamine and 0.5% neomycin. The supernatant containing the viral particles was harvested and clarified by centrifugation (10,000 r.p.m.; 20 min; 4 °C: Beckmann JA 14) 48 h post-electroporation. E protein in the cell culture supernatant was quantified by ELISA, as described below.

### Production of immature virus-containing cell culture supernatant

As controls for maturation ELISAs and specific infectivities, TBEV strain Neudörfl [27] was grown in chicken fibroblasts as described previously [28]. Briefly, 24 h after infection, the cell culture supernatant was harvested, clarified by centrifugation (30 min, 10,000 g, 4 °C), and aliquots were stored at −80 °C (TBEV WT mat). Fresh medium containing 20 mM NH_4_Cl was added to the infected cells, which was left for 1 h and then again replaced with NH_4_Cl-containing medium. Forty-eight hours after infection, the cell culture supernatant was harvested, clarified by centrifugation (30 min, 10,000 g, 4 °C), and aliquots were stored at −80 °C (TBEV WT immat). E protein in the cell culture supernatant was quantified by ELISA, as described below.

### Quantification of E protein by ELISA

E protein concentrations were determined by a four-layer ELISA as described previously [18,29]. Briefly, microtitre plates (Nunc Maxisorp) were coated with a TBEV-specific polyclonal guinea pig serum (2.5 µg ml^−1^) in carbonate coating buffer (15 mM sodium carbonate, 35 mM sodium hydrogen carbonate and pH 9.6) for 48 h at 4 °C. Samples and an internal standard (purified TBEV [30]) were incubated in the presence of 0.4% SDS for 30 min at 65 °C [28]. Serial dilutions of the samples and standard were applied to the coated plates and incubated for 90 min at 37 °C. As primary antibody, a polyclonal rabbit anti-TBEV serum [29] and as secondary antibody a peroxidase-conjugated donkey anti-rabbit immunoglobulin G (DAR-POX, Amersham) were used. Protein concentrations were calculated with the software Gen5 Data Analysis.

### Quantification of RNA copies by quantitative PCR (qPCR)

RNA was isolated from cell culture supernatants with the RNeasy Cleanup system (Qiagen) according to the manufacturer’s instructions. Isolated RNA was then quantified by reverse transcription qPCR as described previously [19,31, 32]. Briefly, cDNA transcription was performed using the iScript cDNA synthesis kit (Bio-Rad) according to the manufacturer’s instructions. Quantitative reverse transcription PCR was carried out with the TaqMan Universal PCR Master Mix (Applied Biosystems).

### Focus assay

Focus assays were performed as described previously [25,32]. Briefly, a monolayer of BHK-21 cells was infected with cell culture supernatants or virus-containing peak fractions. Two days after infection, cells were fixed with 4% paraformaldehyde, and foci were stained with a polyclonal rabbit anti-TBEV serum. As a secondary antibody, an alkaline phosphatase-labelled goat anti-mouse IgG (Sigma-Aldrich) was used, and SigmaFast Fast Red TR/naphthol AS-MX (Sigma-Aldrich) served as substrate.

### Maturation ELISA

The maturation state of WT and mutant particles was determined as described previously [33]. Microtitre plates (Nunc Maxisorp) were coated with a TBEV-specific polyclonal guinea pig serum (2.5 µg ml^−1^) in carbonate coating buffer. After blocking with PBS containing 1% BSA, the serial dilutions of viral or SVPs were added. Monoclonal antibodies (1 µg ml^−1^) B4 (targeting E [34]) or 8H1 (targeting prM [35]) were added and detected via horseradish peroxidase-conjugated rabbit anti-mouse antibody (Nordic Immunology). Mature and immature TBEV and SVPs were used as controls. For each sample, the ratio of 8H1 to B4 of the area under the curve (AUC) was calculated (GraphPad Prism 10).

### In vitro furin cleavage

SVPs were incubated with and without recombinant furin (1.0 U µl^−1^; Enzo Life Sciences) under acidic conditions (pH 5.5) in 150 mM 2MES, 5 mM CaCl2 and 50 mM NaCl for 16 h at 30 °C to allow proteolytic cleavage. Samples were back-neutralized to physiological pH using 150 mM triethanolamine and 10 mM NaCl and subsequently analysed using the maturation ELISA (see above).

### Statistical analyses

Statistical comparisons to the control (WT mature, WT mat) were performed using unpaired t-tests followed by the Holm–Sidak post hoc test to correct for multiple comparisons. For all analyses, measurements were analysed on their original scale and were not normalized to WT. To normalize data distributions, values were log-transformed when indicated. Statistical analyses were performed using GraphPad Prism (version 10), with *P*<0.05 considered statistically significant. In addition, the correlation between mean infectivity and AUC was plotted for each group to visualize the relationship between maturity and infectivity.

## Results

### Identification of tick-borne orthoflavivirus-specific conserved histidine residues in prM and E proteins

Phylogenetically, tick-borne orthoflaviviruses have been divided into three groups: the mammalian tick-borne virus group, the seabird tick-borne virus group and the Kadam tick-borne virus group [36] (Fig. 2a). The mammalian tick-borne orthoflaviviruses include several human pathogens, like TBEV, Omsk haemorrhagic fever virus, Kyasanur Forest disease virus and Powassan virus [36].

Alignments of the amino acid sequences of prM and E proteins revealed six histidine residues that are conserved in tick-borne orthoflaviviruses (Fig. 2b), but not in mosquito-borne orthoflaviviruses. These six residues are strictly conserved across mammalian tick-borne orthoflaviviruses (E-H5, E-104, E-208, E-229, E-419 and prM-H117/M-H28), but only two of them are conserved across all three groups of tick-borne orthoflaviviruses (E-H5, E-H229) (Fig. 2b). Of special interest is the location of E-H208. Mammalian tick-borne orthoflaviviruses possess an insert in the fg loop of E domain II (DII) that is neither present in the other two tick-borne orthoflavivirus groups nor in mosquito-borne orthoflaviviruses, and E-H208 is part of this insert (Fig. 2c).

### Mutagenesis of tick-borne orthoflavivirus-specific conserved histidine residues in prM and E of SVPs

To assess the role of the six histidine residues conserved across mammalian tick-borne orthoflaviviruses (Fig. 2b) in maturation of TBEV, we performed a mutational analysis with SVPs by replacing each histidine with alanine (E-H5A, E-H104A, E-H208A, E-H229A, E-H419A and prM-H117A). As shown previously, these SVPs, which are assembled in eukaryotic cells by co-expression of prM and E proteins, are excellent tools for studying conformational changes of orthoflavivirus envelope proteins during maturation and fusion. They contain the surface proteins in a lipid membrane, undergo the same maturation pathway and exhibit similar fusion characteristics as infectious virions [10,22, 37].

**Fig. 3.**
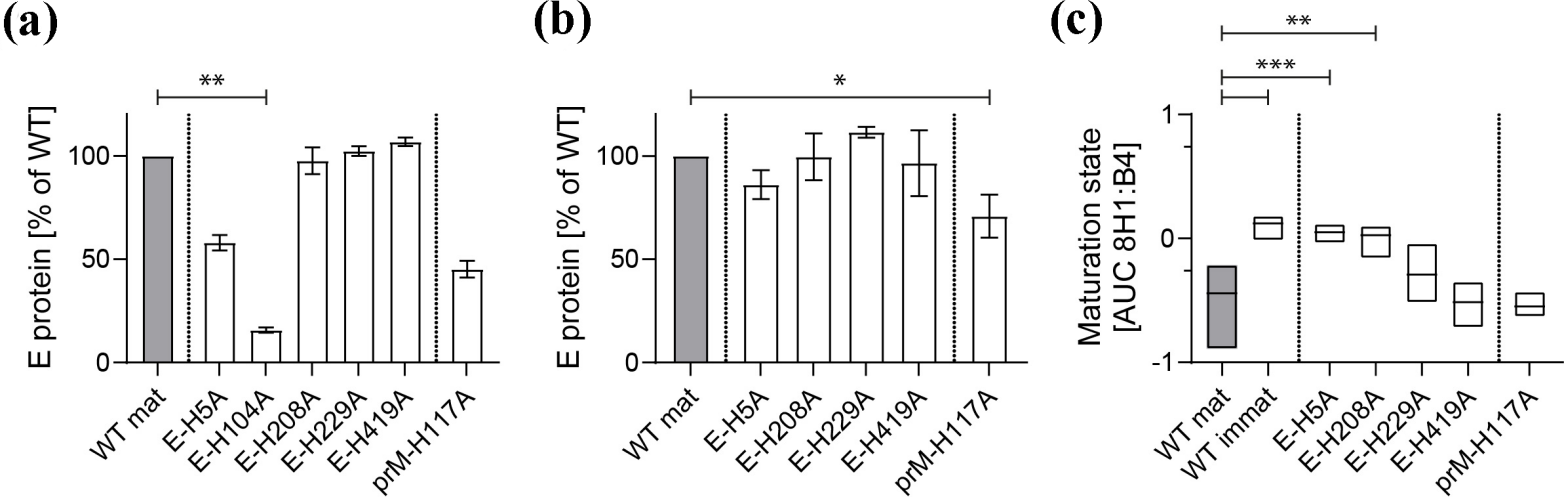
Characterization of WT and mutant recombinant SVPs. (**a**) Secretion of E into the cell culture supernatant of COS-1 cells as determined by a quantitative ELISA. Results are expressed as % E relative to WT. (**b**) Analysis of the particulate nature of secreted E. Samples were subjected to ultracentrifugation and the amount of E in the pellet was determined by quantitative ELISA. Results are expressed as % E relative to WT. (**c**) Analysis of maturation state by ELISA with a prM-specific (8H1) and an E-specific mab (B4). Results are expressed as the ratio of the AUC obtained with mab 8H1 relative to the one obtained with mab B4. Data represent three independent experiments, each performed in technical triplicates; WT mat from the respective individual productions was included as a control in each experiment (*n*=12); error bars represent the standard errors of the means. Asterisks indicate significant differences to WT mat. Comparisons to control (WT mat) were assessed by unpaired t-tests with Holm–Sidak post hoc adjustment (***,*P*<0.001; **P<0.01; *P<0.05). mat, mature; immat, immature.

After transfection of COS-1 cells with the respective plasmids, the cell-culture supernatant was harvested 48 h after electroporation, and SVP secretion was analysed by quantifying E in the cell culture supernatant with an ELISA, as described in Methods (Fig. 3a). Their particulate state was assessed by ultracentrifugation-based separation of particle-associated and soluble fractions (Fig. 3b). The amount of E protein in both fractions was determined by a quantitative ELISA (see Methods). The E-H5A and prM-H117A mutants had reduced levels of E in the cell culture supernatant compared to the WT (Fig. 3a). In the case of the E-H104A mutant, almost no E protein could be detected, indicating impaired protein expression, assembly and/or secretion (Fig. 3a). We therefore had to exclude this variant from further experiments. The residual five mutants were able to form secreted particles like the WT (Fig. 3b).

To analyse the maturation state of the secreted mutant SVPs, we determined their prM content by an ELISA with monoclonal antibodies (mabs) recognizing prM (8H1) and E (B4). We included an immature control obtained by production of SVPs in the presence of NH_4_Cl, which raises the intracellular pH and thus inhibits prM cleavage [22]. As shown in Fig. 3(c), the E-H5A and E-H208A mutants had a similar prM content as the immature WT control, indicating an impairment of prM processing. In contrast, the E-H229A, E-H419A and prM-H117A mutants were not significantly different from the mature WT control. The E-H229A mutant showed characteristics consistent with impaired or heterogeneous maturation.

**Fig. 4.**
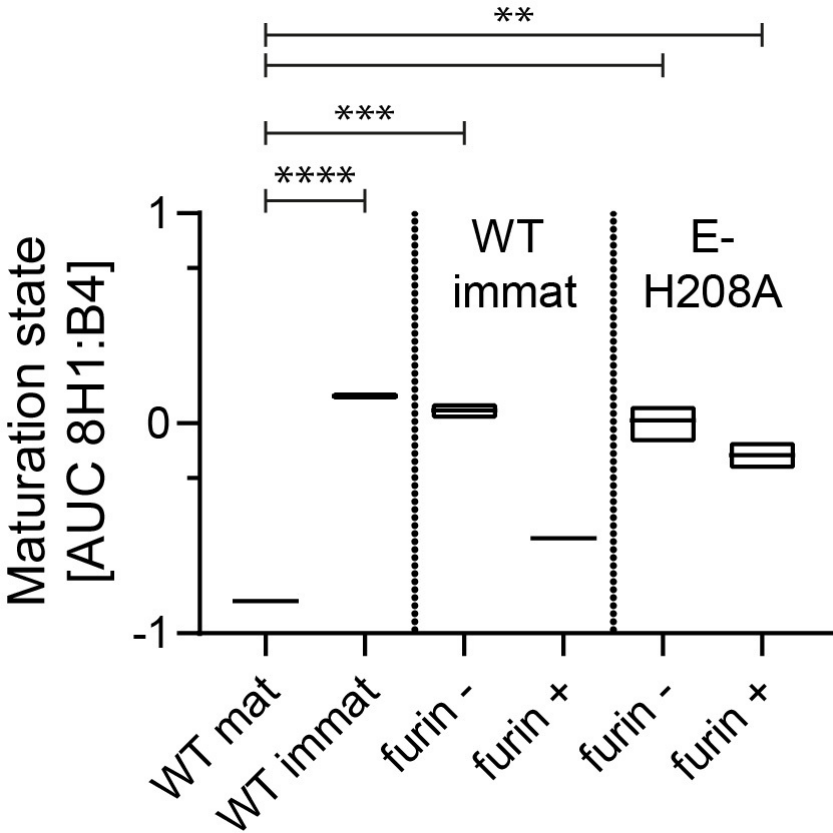
*In vitro* furin cleavage of WT and E-H208A SVPs. SVPs were incubated under acidic conditions (pH 5.5) in the presence (1 U) or absence (0 U) of recombinant furin, and back-neutralized, and the maturation state was analysed by ELISA with a prM-specific (8H1) and an E-specific mab (B4). Mature and immature WT SVPs were included as ELISA controls. Results are expressed as the ratio of the AUC obtained with mab 8H1 relative to the one obtained with mab B4. Data are from three independent experiments; error bars represent the standard errors of the means. Asterisks indicate significant differences to WT mat. Comparisons to control (WT mat) were assessed by unpaired t-tests with Holm–Sidak post hoc adjustment (****P<0.0001; ***P<0.001; **P<0.01). mat, mature; immat, immature.

To confirm the assumed impairment of prM cleavage in the two mutants E-H5A and E-H208A, we performed *in vitro* experiments with recombinant furin at low pH. After back-neutralization, the furin-treated and untreated samples were analysed by ELISA using the prM- and E-specific mabs 8H1 and B4, respectively. The immature WT control (SVPs produced in NH_4_Cl-treated cells) had a significantly lower AUC ratio (8H1:B4) after furin treatment (Fig. 4), indicating efficient prM cleavage (comparison of furin-treated and untreated WT by paired t-test, **P=0.004). The E-H5A SVPs could not be analysed in *in vitro* cleavage experiments, as they lost reactivity with both mabs (data not shown). E-H208A SVPs remained predominantly immature after furin treatment (Fig. 4, comparison of furin-treated and untreated mutant by paired t-test, not significant, *P*=0.09), supporting the interpretation that the introduced mutation indeed interfered with prM cleavage.

### Mutagenesis of tick-borne orthoflavivirus-specific-conserved histidine residues in prM and E of infectious virus

**Fig. 5.**
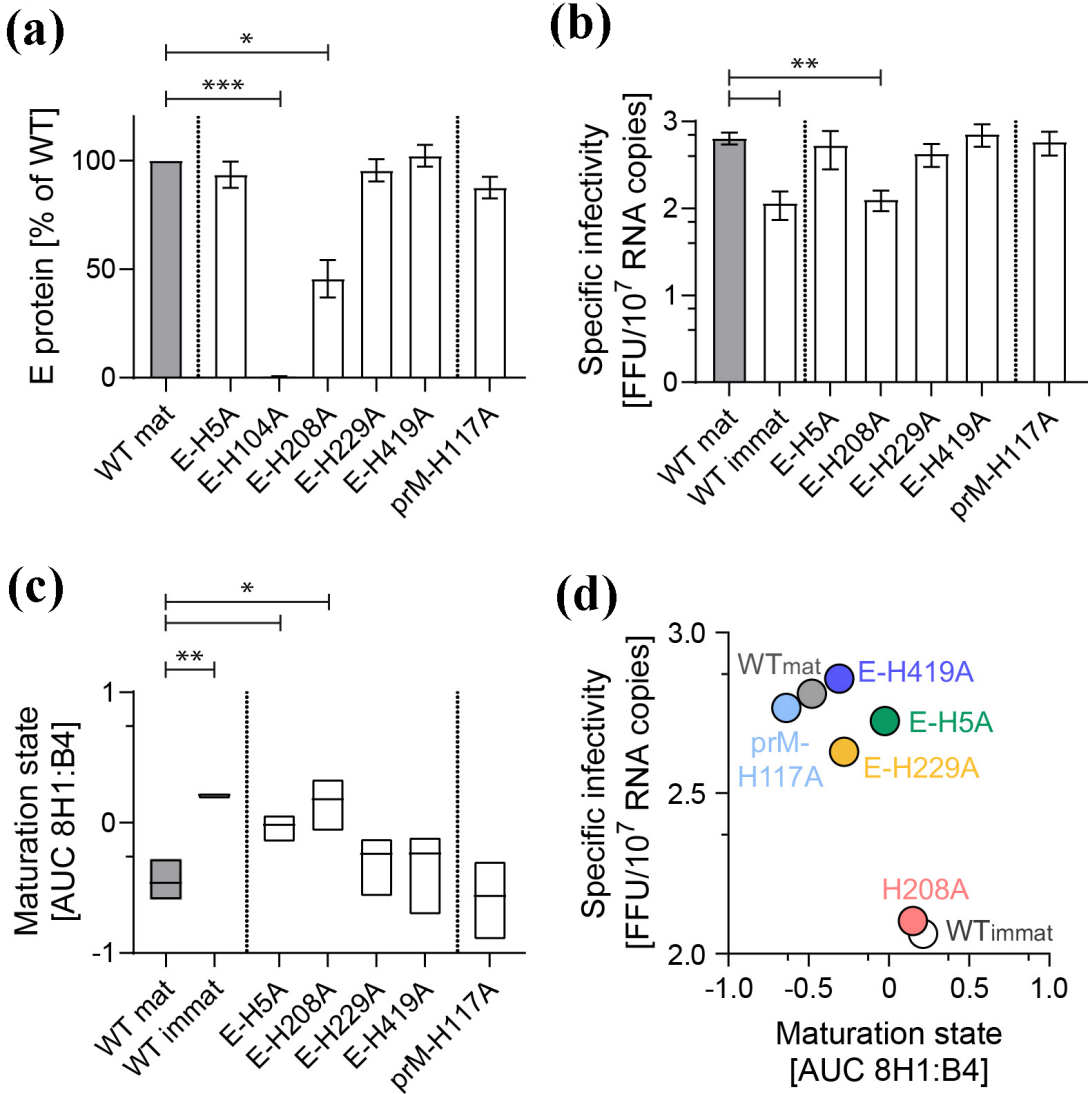
Characterization of WT and mutant viral particles. (**a**) Secretion of E into the cell culture supernatant of BHK cells as determined by a quantitative ELISA. Results are expressed as % E relative to WT. (**b**) Specific infectivity is expressed as f.f.u. normalized to 10⁷ RNA copies (f.f.u. per 10⁷ RNA copies). (**c**) Analysis of maturation state by ELISA with a prM-specific (8H1) and an E-specific mab (B4). Results are expressed as the ratio of the AUC obtained with mab 8H1 relative to the one obtained with mab B4. (**d**) Mapping of specific infectivity, expressed as f.f.u. normalized to 10⁷ RNA copies, in relation to maturation state, as determined by the AUC ratio of prM-specific (8H1) versus E-specific (B4) monoclonal antibody reactivities. Data represent three independent experiments, each performed in technical triplicates; WT mat from the respective individual productions was included as a control in each experiment [*n*=9 for panels (a) and (b); *n*=4 for panel (c)]; error bars represent the standard errors of the means. Asterisks indicate significant differences to WT mat. Comparisons to control (WT mat) were assessed by unpaired t-tests with Holm–Sidak post hoc adjustment (***P<0.001; **P<0.01; *P<0.05). mat, mature; immat, immature.

Since SVPs do not allow studying effects on infectivity, we also introduced the six histidine–alanine mutations into an infectious TBEV clone. *In vitro* transcribed RNAs were transfected into BHK cells, and the cell culture supernatant was harvested 48 h after transfection. Virus secretion was determined by quantifying E in the cell culture supernatant by ELISA (see Methods). As for the SVPs, almost no E protein was detected in the cell culture supernatant of the E-H104A mutant, and therefore, the mutant could not be further analysed (Fig. 5a). A reduction was also observed for the H208A mutant, but WT-like levels of secreted E were found for the E-H5A, E-H229A, E-H419A and prM-H117A mutants (Fig. 5a).

To assess the impact of histidine substitutions on the specific infectivities of mutated viruses, we quantified f.f.u. of the virus-containing cell culture supernatant and related those to viral RNA copy numbers determined by qPCR (Fig. 5b). As shown in Fig. 5(b), the E-H208A mutant exhibited a slightly but significantly lower specific infectivity than the WT (5.1-fold), thus being similar to the immature virus control (5.6-fold reduction). All other mutants were similar to the mature WT. Specific infectivity is shown as infectious units (f.f.u.) normalized to 10⁷ RNA copies. Absolute values of infectious titres (f.f.u. ml^−1^) and genome copies (RNA copies/ml) are provided in Fig. S1 (available in the online Supplementary Material).

The maturation state of the mutated virus particles was analysed by ELISA with mabs recognizing prM (8H1) and E (B4). We included virus grown in the presence of NH_4_Cl as an immature WT control [28]. As shown in Fig. 5(c), the E-H208A mutant had a similar prM content as the immature WT, indicating a strongly impaired prM cleavage during maturation. The prM content of the E-H5A mutant was also significantly higher than that of the mature WT control, but lower than that of the immature WT (Fig. 5c), consistent with impaired or heterogeneous maturation. The E-H229A, E-H419A and prM-H117A mutants were not significantly different from the mature WT control (Fig. 5c), with E-H229A and E-H419A being slightly more immature than the WT and prM-H117A.

To better visualize the properties of the different mutants in comparison to the mature and immature WT preparations, we plotted their specific infectivity versus the maturation state (Fig. 5d). This analysis demonstrates that only the E-208A mutant clustered with the immature control (Fig. 5d). All other mutants clustered with the mature WT control (Fig. 5d).

## Discussion

In this work, we identified a histidine residue (H208) in the TBEV E protein that is absolutely conserved across mammalian tick-borne orthoflaviviruses, but not in other tick-borne and mosquito-borne orthoflaviviruses, as an important distinguishing factor in the maturation of TBEV. Importantly, both maturation and concomitantly specific infectivity of the E-208A TBEV mutant were significantly impaired compared to the mature WT. The replacement of the other tick-borne-conserved histidine residues E-H5, E-H229, E-H419 and prM-H117 by alanine had less impact on virus maturation, and the specific infectivity of these mutants was not affected by the introduced mutations.

**Fig. 6.**
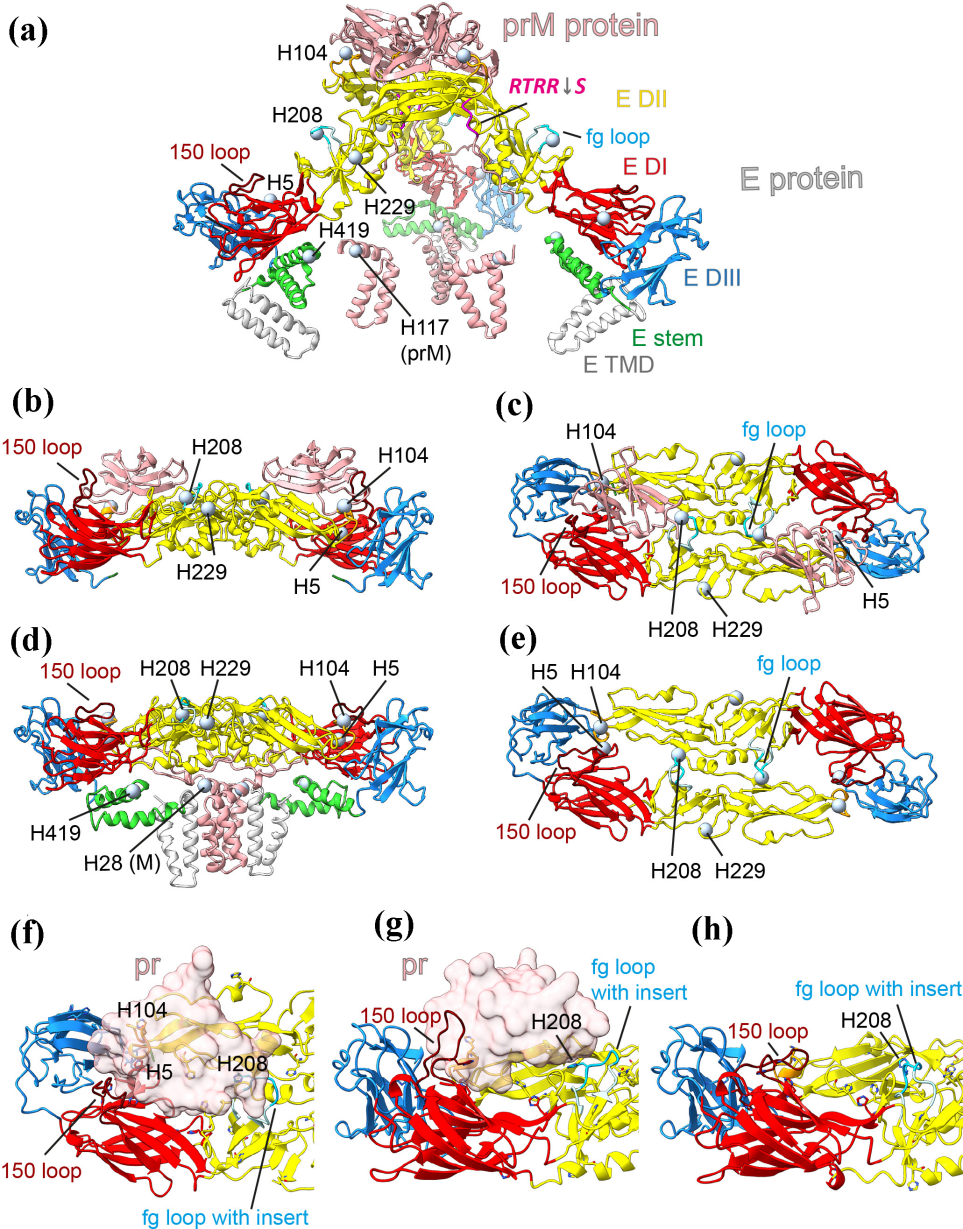
Structures of the TBEV envelope proteins. (**a**) Location of the tick-borne specific histidine residues in the primary assembly product (spiky immature particles in the ER at neutral pH). Cartoon representation of the prM–E trimeric spike in its side view. PDB: 8PPQ [38]. (**b, c**) Location of tick-borne specific histidine residues in low-pH immature particles (smooth immature particles in the secretory pathway at acidic pH). Cartoon representation of the pr–E heterodimer in its side view (**b**) and top view (**c**). PDB: 7QRE [17]. (**d, e**) Location of tick-borne specific histidine residues in mature particles (smooth mature extracellular particles at neutral pH). Cartoon representation of the E homodimer with M in its side view (**d**) and the E homodimer in its top view (**e**). PDBs: 5O6A [16] and 1SVB [45]. (**f, g**) Zoom of the pr–E interaction site in the smooth pr–E dimer in its top view (**f**) and side view (**g**). (**h**) Zoom on the same site as in panel (g), but in the mature E dimer. The structures were visualized with ChimeraX [46]. Colour code prM and E as in Fig. 1. Histidine residues are shown as spheres in panels (a)–(e), as sticks in panels (f)–(h). The fg loop is shown in light cyan and the H208-containing insert in bright cyan. The FCS (RTRR ↓ S) in panel (a) is shown in magenta. E DI, E domain I; E DII, E domain II; E DIII, E domain III.

H208 is located within the immature trimeric (prM–E)_3_ spikes at a site, where it is not involved in prM–E interactions (Fig. 6a). In this conformation, the FCS is occluded (Fig. 6a [38]). After the low-pH-induced conversion into the smooth form [(prM–E)_2_] in the secretory pathway which allows furin cleavage (Fig. 1b), H208 is found at the pr–E interaction site and at the DII-E dimer interface [17] (Fig. 6b, c, g). In the structure of the soluble pr–E heterodimer of TBEV (Fig. 6b, c), the protonated side chain of H208 forms an inter-protomeric salt bridge with D253, which in turn interacts with R98 in pr (Fig. S2A [17]), highlighting the role of H208 in stabilizing this interface. The Missense3D analysis ([39]; https://missense3d.bc.ic.ac.uk/~missense3d2/) predicts that substituting H208 by alanine induces a local structural change at this site (Fig. S2B, C), consistent with altered packing, while no major changes are predicted in the immature spiky or mature smooth conformations. Thus, the H208A mutation may impair the adoption of a prM cleavage-competent conformation during maturation, providing a structural basis for the observed immature phenotype of the E-H208A mutant.

The specific infectivity of the immature E-H208A mutant was reduced by 5.1-fold compared to the mature WT control virus, similar to the reduction observed for immature virus generated in NH_4_Cl-treated cells (5.6-fold), meaning that these particles still possess considerable infectivity. These findings are consistent with the specific infectivities of TBEV and other tick-borne orthoflaviviruses grown in furin-deficient LoVo cells, which generate virions with completely uncleaved prM [13], but show a similar decrease in specific infectivity (<10-fold compared to the mature virus) [13]. In contrast, mosquito-borne orthoflaviviruses grown in LoVo cells had a significantly lower specific infectivity, at least 1,000-fold compared to mature controls [13,40]. This difference was explained to be due to the irreversible low-pH-induced exposure of the FCS in tick-borne orthoflaviviruses, which allows furin located in the cell membrane of new cells to cleave prM, thereby enabling efficient virus entry [13]. In mosquito-borne orthoflaviviruses, the low-pH-dependent transition is reversible, making activation during infection less likely.

All of these immature TBEV forms (virus grown in NH_4_Cl-treated cells or LoVo cells, E-H208A mutant) have a relatively high specific infectivity, but they differ in important details: LoVo cell-grown TBEV has been exposed to acidic pH in the secretory pathway and may therefore be in a conformation with the FCS exposed, enabling prM processing after cell release. In contrast, TBEV grown in NH_4_Cl-treated cells has not transited through acidic compartments and should therefore remain in the spiky conformation, in which the FCS is hidden. TBEV preparations grown in NH_4_Cl-treated cells, however, were shown to exhibit some structural heterogeneity [38]. Although most of the virus particles were spiky, some partially mature virus particles were present. Such particles were shown to be able to infect cells without the need for activation by furin-like proteases [41], explaining their relatively high specific infectivity. This may explain why the E-H208A mutant clusters with the immature WT in Fig. 5(d). The E-H208A mutant, however, has been exposed to both low pH and furin, and prM cleavage was impaired due to the introduced mutation (Figs 3, 4 and 5). The observed infectivity of this mutant might be caused by either a mixture of immature and mature particles or the presence of partially mature particles. To find out how the infectivity of the E-H208A mutant can be explained, information on the structural conformation of the mutant would be necessary.

The mutants E-H5A, E-H229A, E-H419A and prM-H117A were more similar to the mature WT than to the immature WT (Fig. 5d), indicating that protonation/deprotonation of these histidine residues are neither essential for maturation nor for fusion. We cannot exclude, however, that these particles fuse at a lower pH in late endosomes or are important for the life cycle in tick cells but not in mammalian cells. Some impairment of prM cleavage was observed, particularly for the E-H5A mutant, although its specific infectivity was unaffected (Fig. 5b). H5, located at the N-terminus of E, is positioned at the side of the (prM–E)₃ spike and is not involved in prM–E interactions (Fig. 6a). Protonation of H5 at acidic pH in the TGN likely contributes to the formation of an open conformation of the 150 loop (Fig. 6g), facilitating proper positioning of the pr domain and efficient prM cleavage [17], and its removal may interfere with this process. However, the intrinsic positive charge of the N-terminus and additional histidine residues [17] likely compensate for the absence of H5, preserving infectivity.

The E-H104A mutant could not be investigated, because viral proteins were either not expressed or particles were not formed and/or secreted. H104 participates in E-prM interactions in both immature spiky (Fig. 6a) [38] and immature smooth particles (Fig. 6b, c, f) [17], and its disruption might impair prM–E heterodimer formation, particle assembly and/or maturation. Notably, H104 distinguishes tick-borne from all other orthoflaviviruses, which harbour a glycine at this position, including mosquito-borne, no-known-vector insect-specific tick-associated and aquatic viruses, suggesting a specific adaptation of the tick-borne group (Fig. S3). Among these, Kadam virus uniquely encodes an asparagine, although its significance remains unclear (Figs 2c and S3).

The H208-containing insert (Fig. 2b) is unique to mammalian tick-borne orthoflaviviruses and neither present in seabird and Kadam tick-borne, mosquito-borne, no-known vector and insect-specific orthoflaviviruses. As mammalian tick-borne orthoflaviviruses are considered to have evolved more recently than the seabird and Kadam groups [36,42], this insert was possibly acquired through adaptation to mammalian hosts and/or specific tick vectors. The insert provides additional prM–E/E–E interactions in smooth immature particles as well as E–E interactions in mature particles (Fig. 6g, h), which might necessitate the presence of an additional histidine residue to control low-pH-induced structural changes in the viral life cycle, such as the exposure of the FCS necessary for acquiring membrane fusion activity. The E dimer interactions provided by H208 and the insert in mature virions might also contribute to the increased stability of TBEV E dimers lacking the stem-anchor region (soluble E, sE) compared to mosquito-borne orthoflaviviruses [17,43, 44]. A comparison of high-resolution structures of mammalian tick-borne orthoflavivirus sE dimers shows a similar positioning of H208 within the E dimer interface (Fig. S4), supporting a conserved structural role of this residue and its broader relevance across this group.

Taken together, our findings demonstrate that tick-borne orthoflaviviruses have evolved unique histidine residues, setting them apart from other orthoflaviviruses. We have shown that one of these residues (H208) is essential for TBEV maturation and that these immature E-H208A mutant particles are almost as infectious as the WT, consistent with comparative analyses of tick-borne and mosquito-borne orthoflaviviruses [13]. Tick-borne orthoflavivirus-conserved histidine residues likely represent an adaptation to specific hosts and/or vectors, thus being an expression of the particular requirements of the complex ecological life cycle of this group of viruses.

## Supplementary material

10.1099/jgv.0.002286 Fig. S1.

## Abbreviations

AUC: area under the curve
DII: domain II
DMEM: Dulbecco’s modified Eagle’s medium
E: envelope
ER: endoplasmic reticulum
FCS: furin cleavage site
mab(s): monoclonal antibody/antibodies
MEM: minimum essential medium
prM/M: pre-membrane/membrane
rf: restriction-free
SVP(s): subviral particle(s)
TBEV: tick-borne encephalitis virus
