# Supplementary material for: Conserved histidine residues and the control of tick-borne encephalitis virus maturation
Source: J Gen Virol. 2026 Jun 18;107(6):002286. doi: 10.1099/jgv.0.002286 (PMC13278375; doi:10.1099/jgv.0.002286)
Supplement: Fig. S1. [file jgv-107-02286-s001.pdf]

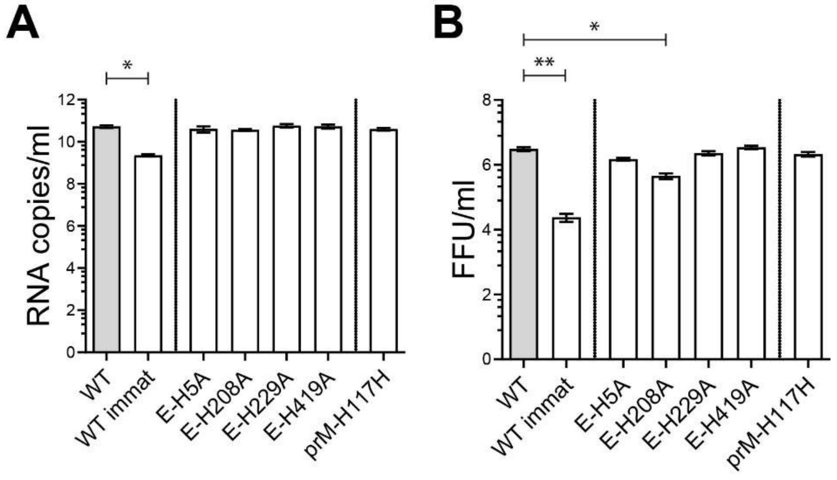

Supplementary Figure S1. Viral RNA levels and infectious titers of WT and mutant viruses.
(A) Viral genome copies (RNA/ml) in cell culture supernatants, quantified by RT-qPCR. (B) Infectious titers (FFU/ml) determined by focus-forming assay. Data represent three independent experiments, each performed in technical triplicates; WTmat from the respective individual productions was included as a control in each experiment (n = 9); error bars represent the standard errors of the means. Asterisks indicate significant differences to WT mat. Comparisons to control (WT mat) were assessed by unpaired t-tests with Holm-Sidak post-hoc adjustment (**, p<0.01; *, p<0.05). FFU, focus-forming units; mat, mature; immat, immature; WT, wild type.

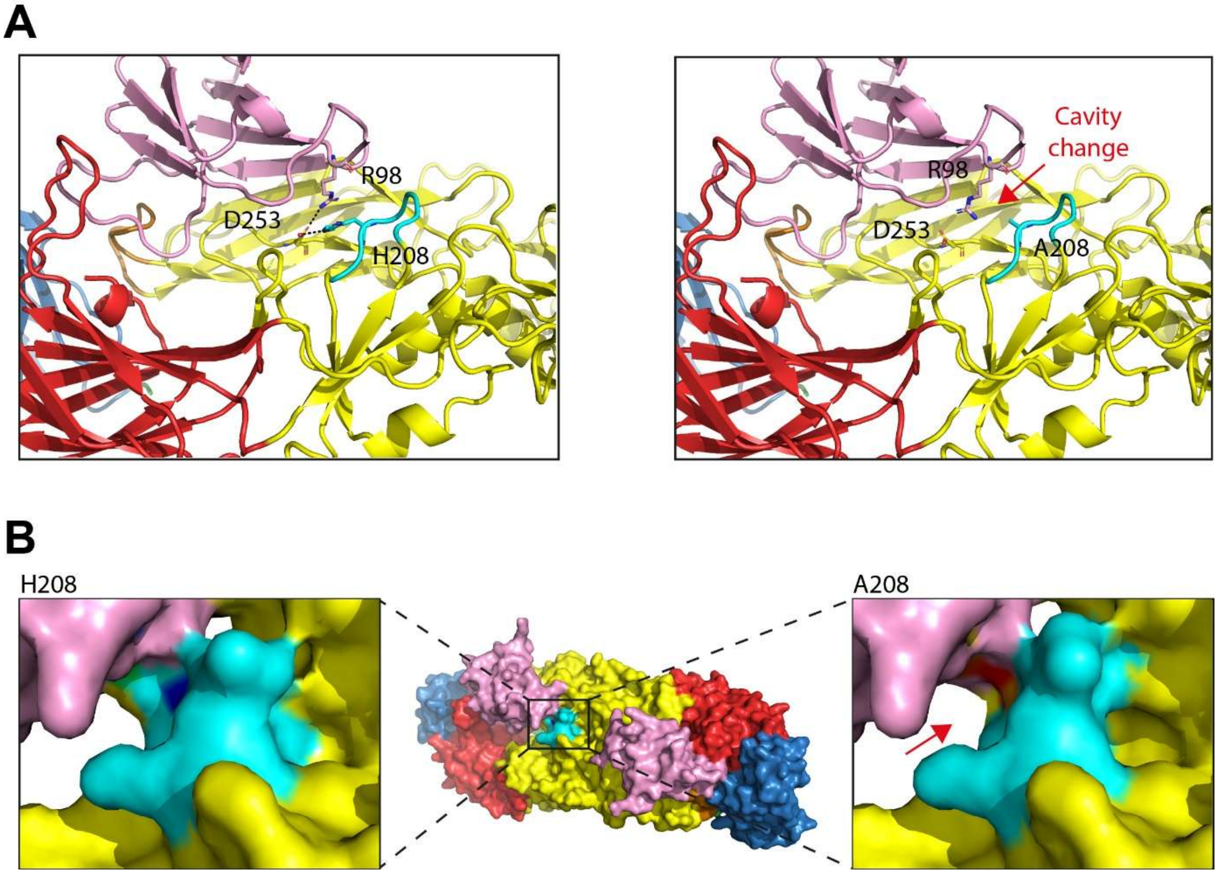

Supplementary Figure S2. Structural impact of the H208A substitution on the TBEV pr–E heterodimer (PDB: 7QRE) predicted by Missense3D (https://missense3d.bc.ic.ac.uk/~missense3d2/).

(A) Cartoon representations of the TBEV pr–E heterodimer showing the local environment of residue 208 in the wild-type (H208, left) and alanine mutant (A208, right). Residue 208 and interacting residues (D253, R98) are shown as sticks. (B) Surface representation of the pr–E heterodimer highlighting the local environment of residue 208. The fg loop containing H208 (left) or A208 (right) is shown in cyan. The central panel indicates the position of residue 208 within the overall structure. The H208A substitution results in a local expansion of cavity volume, which is indicated by the red arrow. In the wild-type, H208 forms interactions with D253 and neighboring residues.

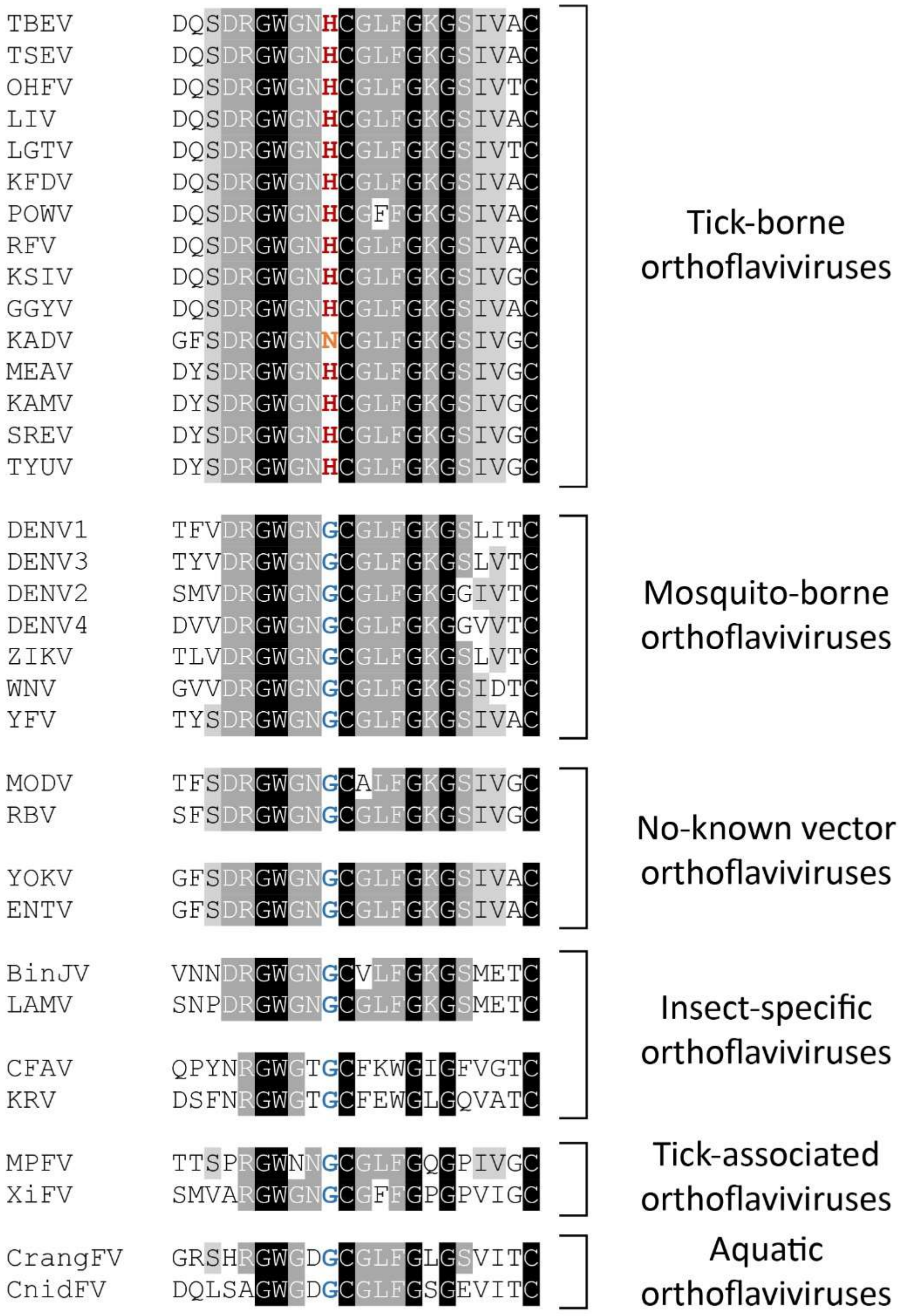

Supplementary Figure S3. Amino acid alignment of the fusion loop of different orthoflaviviruses. Residues 95-116 (TBEV numbering). GenBank accession numbers: TBEV, tick-borne-encephalitis virus (U27495); TSEV, Turkish sheep encephalitis virus (DQ235151); LIV, louping Ill virus (NC_001809); OHFV, Omsk hemorrhagic fever virus (AY193805); LGTV, Langat virus (AF253419); KFDV, Kyasanur forest disease virus (AY323490); POWV, Powassan virus (L06436); KSIV, Karshi virus (DQ235147); GGYV, Gadgets Gully virus (DQ235145); RFV, Royal farm virus (DQ235149); KAMV, Kama virus (KF815940); MEAV, Meaban virus (DQ235144); SREV, Saumarez Reef virus (DQ235150); TYUV, Tyuleniy virus (DQ235148); KADV, Kadam virus (DQ235146); DENV1, dengue virus serotype 1 (GQ398255); DENV3, dengue virus serotype 3 (EU081190); DENV2, dengue virus serotype 2 (NC_001474); DENV4, dengue virus serotype 4 (GQ398256); ZIKV, Zika virus (KJ776791); WNV, West Nile virus (DQ211652); YFV, yellow fever virus (AY640589); MODV, Modoc virus (NC_003635); RBV, Rio Bravo virus (NC_003675); YOKV, Yokose virus (AB114858); ENTV, Entebbe bat virus (AY632537); BinJV, Binjari virus (MG587038); LAMV, Lammi virus (FJ606789); CFAV, Cell fusing agent virus (M91671); KRV, Kamiti river virus (AY149904); MPFV, Mpulungu flavivirus (LC582740); Xinyang flavivirus, XiFV, (OP699738); CrangFV, Crangon crangon flavivirus (MK473878); CnidFV, Cnidaria flavivirus (OX394137).

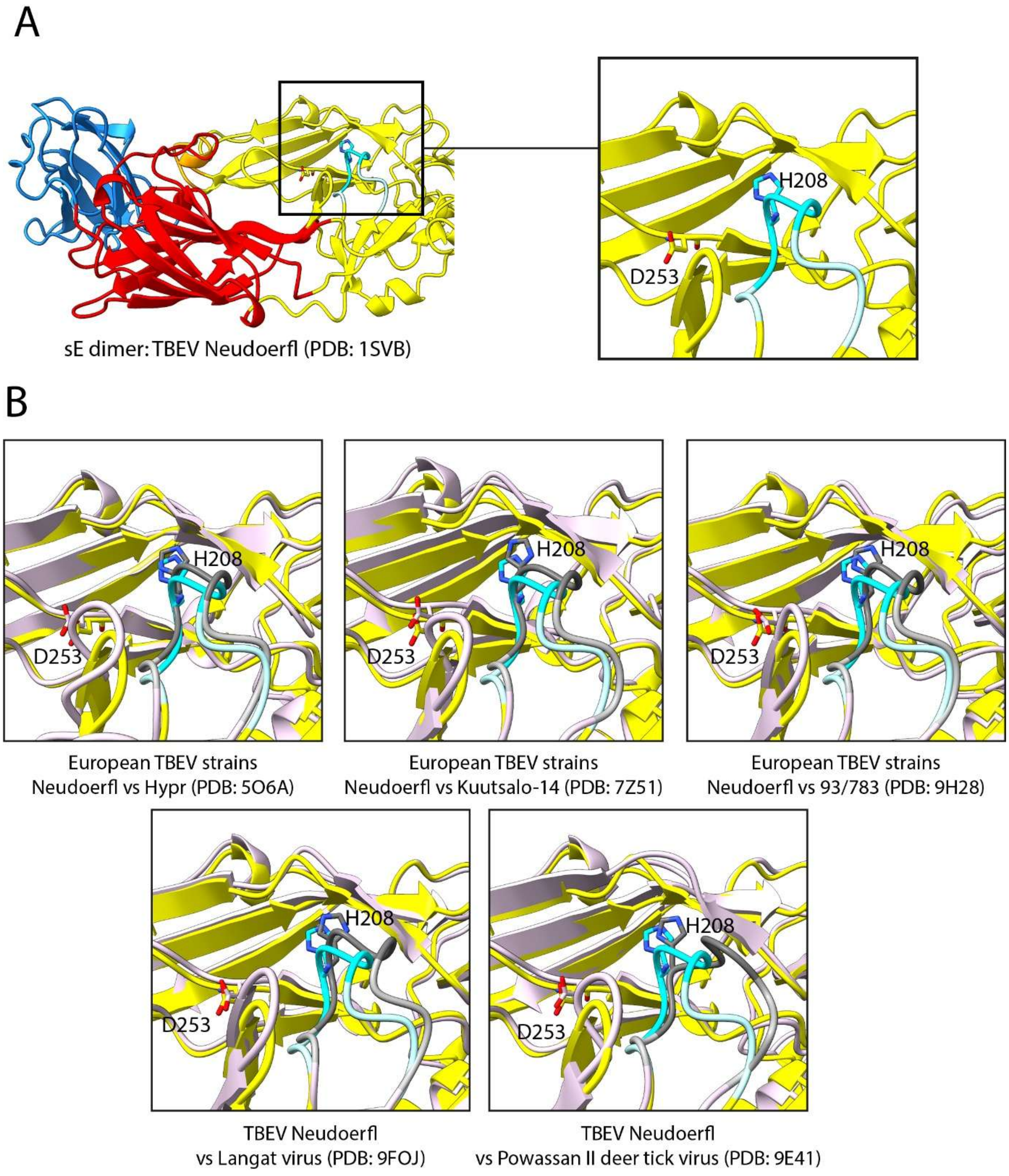

Supplementary Figure S4. H208 in different high-resolution structures of sE of mammalian tick-borne orthoflaviviruses.

(A) Cartoon representation of the TBEV Neudoerfl sE dimer (PDB: 1SVB): H208 and D253 do not form a salt bridge as in the pr-sE heterodimer (compare with Fig. S2).

(B) Enlargement of the framed region in (A) showing sE dimers of different tick-borne orthoflaviviruses in comparison with the TBEV Neudoerfl sE dimer. TBEV Neudoerfl is colored as in (A), with the fg loop in light cyan and the H208-containing insert in cyan. Other tick-borne orthoflaviviruses are shown in light purple, with the fg loop in light grey and the H208-containing insert in grey.

H208 and D253 are shown as sticks. Virus names and corresponding PDB codes are indicated below the structures.
